# (*E*)-1-(2-Nitro­benzyl­idene)-4-phenyl­thio­semicarbazide

**DOI:** 10.1107/S1600536812026803

**Published:** 2012-06-20

**Authors:** Samaneh Feizi, Ali Hossein Rezayan, Soroush Sardari, Behrouz Notash

**Affiliations:** a Drug Design and Bioinformatics Unit, Medical Biotechnology Department, Biotechnology Research Center, Pasteur Institute, Tehran Iran13164; b Department of Life Science Engineering, Faculty of New Science and Technology, University of Tehran, Tehran, Iran; c Department of Chemistry, Shahid Beheshti University, G. C., Evin, Tehran, 1983963113, Iran

## Abstract

In the title mol­ecule, C_14_H_12_N_4_O_2_S, the conformation about the imine bond is *trans*. The dihedral angle between the two rings is 88.22 (11)°. An intra­molecular N—H⋯N contact occurs. The crystal structure features N—H⋯S and C—H⋯O hydrogen bonds.

## Related literature

For background to thio­semicarbazone derivatives, see: Shaabani *et al.* (2011 ▶); Sardari *et al.* (1999 ▶). For applications of imine bonds in synthesis, see: Plech *et al.* (2011 ▶); Tada *et al.* (2011 ▶); Sriram *et al.* (2007 ▶). For related structures, see: Jian & Li (2006*a* ▶,*b* ▶); Fang *et al.* (2007 ▶).
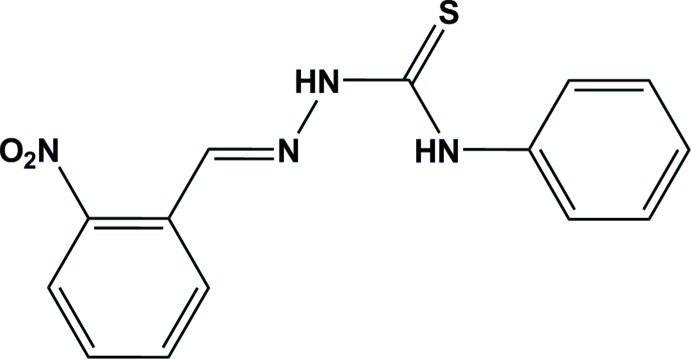


## Experimental

### 

#### Crystal data

C_14_H_12_N_4_O_2_S
*M* *_r_* = 300.35
Triclinic, 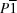

*a* = 7.4050 (7) Å
*b* = 8.4239 (8) Å
*c* = 12.2363 (10) Å
α = 90.382 (7)°
β = 93.974 (7)°
γ = 110.004 (8)°
*V* = 715.14 (12) Å^3^
*Z* = 2
Mo *K*α radiation
μ = 0.24 mm^−1^
*T* = 298 K
0.38 × 0.35 × 0.32 mm

#### Data collection

Stoe IPDS II diffractometer
Absorption correction: numerical (*X-RED* and *X-SHAPE*; Stoe & Cie (2005 ▶) *T*_min_ = 0.910, *T*_max_ = 0.930
7967 measured reflections
3832 independent reflections
2715 reflections with *I* > 2σ(*I*)
*R*_int_ = 0.043

#### Refinement

*R*[*F* ^2^ > 2σ(*F* ^2^)] = 0.053
*wR*(*F* ^2^) = 0.128
*S* = 1.03
3832 reflections
202 parameters
H atoms treated by a mixture of independent and constrained refinement
Δρ_max_ = 0.26 e Å^−3^
Δρ_min_ = −0.23 e Å^−3^

### 

Data collection: *X-AREA* (Stoe & Cie, 2005 ▶); cell refinement: *X-AREA*; data reduction: *X-AREA*; program(s) used to solve structure: *SHELXS97* (Sheldrick, 2008 ▶); program(s) used to refine structure: *SHELXL97* (Sheldrick, 2008 ▶); molecular graphics: *ORTEP-3 for Windows* (Farrugia, 1997 ▶); software used to prepare material for publication: *WinGX* (Farrugia, 1999 ▶).

## Supplementary Material

Crystal structure: contains datablock(s) I, global. DOI: 10.1107/S1600536812026803/bt5899sup1.cif

Structure factors: contains datablock(s) I. DOI: 10.1107/S1600536812026803/bt5899Isup2.hkl

Supplementary material file. DOI: 10.1107/S1600536812026803/bt5899Isup3.cml

Additional supplementary materials:  crystallographic information; 3D view; checkCIF report

## Figures and Tables

**Table 1:** Hydrogen-bond geometry (Å, °)

*D*—H⋯*A*	*D*—H	H⋯*A*	*D*⋯*A*	*D*—H⋯*A*
N3—H3*B*⋯S1^i^	0.87 (2)	2.51 (2)	3.3664 (18)	168.8 (19)
N4—H4*B*⋯N2	0.79 (3)	2.29 (2)	2.642 (2)	108 (2)
C11—H11⋯O1^ii^	0.93	2.60	3.214 (3)	125

Symmetry codes: (i) 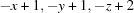; (ii) 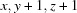.

**Fig. 1.**
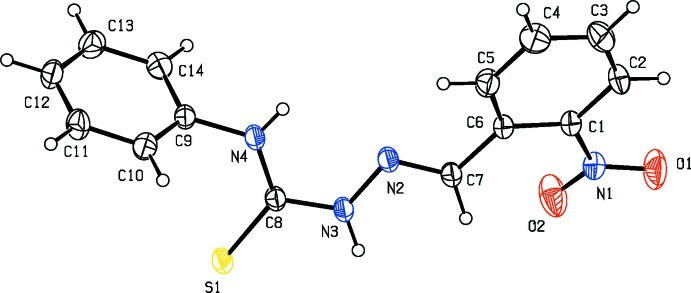
The asymmetric unit of the title compound with displacement ellipsoids drawn at 30% probability level.

**Fig. 2.**
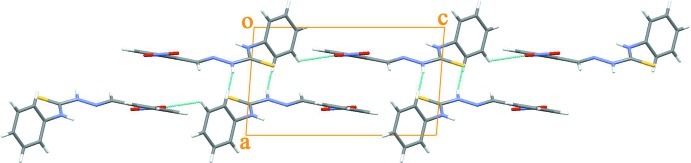
The packing diagram of the title compound. The intermolecular N—H···S and C—H···O hydrogen bonds are shown as blue dashed lines.

## supplementary crystallographic information

### Comment

Thiosemicarbazone derivatives are of special importance because of their versatile biological and pharmacological activities. Thiosemicarbazides are potent intermediates for the synthesis of pharmaceutical and bioactive materials and thus, they are used extensively in the field of medicinal chemistry. The imine bond (–N=CH–) in this compounds are useful intermediates in organic synthesis, in particular for the preparation of heterocycles and non-natural β-aminoacids (Plech *et al.*, 2011; Tada *et al.*, 2011; Sriram *et al.*, 2007).

In a continuation of our research on the development of synthetic methods in heterocyclic chemistry (Shaabani *et al.*, 2011; Sardari *et al.*, 1999) here we report the synthesis and structure of the title compound.

The asymmetric unit of the title compound is shown in Fig. 1. In the title molecule, the configuration is *trans* about the imine bond (C=N). Bond lengths and angles are in the normal ranges reported for similar structures (Jian and Li (2006**a*,b*); Fang *et al.* (2007)). The dihedral angle between the two phenyl ring is 88.22 (11)°. The crystal structure exhibits intermolecular N—H···S and C—H···O hydrogen bonds and also intramolecular N—H···O and C—H···N hydrogen bonds (Fig. 2 & Table 1).

### Experimental

To a magnetically stirred solution of 4-phenylthiosemicarbazide (0.167 g, 1.0 mmol) in MeOH (30 ml) in round bottom flask was added a solution of 2-nitrobenzaldehyde (0.151 g, 1 mmol) at room temperature. The mixture was stirred for 48 h. After completion of the reaction, the Precipitate product was filtered and washed with MeOH (20 ml) and dried at room temperature. The final product is a yellow solid (yield 70%).

### Refinement

The hydrogen atom attached to nitrogen atoms and C—H of imine moiety were found in difference Fourier map and refined isotropically without restraint. Aromatic C—H protons were positioned geometrically and refined as riding atoms with C—H = 0.93 Å and *U*iso(H) = 1.2 *U*eq(C).

### Crystal data

**Table:**

C_14_H_12_N_4_O_2_S	*Z* = 2
*M**_r_* = 300.35	*F*(000) = 312
Triclinic, *P*1	*D*_x_ = 1.395 Mg m^−^^3^
Hall symbol: -P 1	Mo *K*α radiation, λ = 0.71073 Å
*a* = 7.4050 (7) Å	Cell parameters from 7967 reflections
*b* = 8.4239 (8) Å	θ = 2.6–29.2°
*c* = 12.2363 (10) Å	µ = 0.24 mm^−^^1^
α = 90.382 (7)°	*T* = 298 K
β = 93.974 (7)°	Prism, yellow
γ = 110.004 (8)°	0.38 × 0.35 × 0.32 mm
*V* = 715.14 (12) Å^3^	

### Data collection

**Table:**

Stoe IPDS II diffractometer	3832 independent reflections
Radiation source: fine-focus sealed tube	2715 reflections with *I* > 2σ(*I*)
Graphite monochromator	*R*_int_ = 0.043
Detector resolution: 0.15 mm pixels mm^-1^	θ_max_ = 29.2°, θ_min_ = 2.6°
rotation method scans	*h* = −10→10
Absorption correction: numerical (*X-RED* and *X-SHAPE*; Stoe & Cie (2005)	*k* = −11→10
*T*_min_ = 0.910, *T*_max_ = 0.930	*l* = −15→16
7967 measured reflections	

### Refinement

**Table:**

Refinement on *F*^2^	Primary atom site location: structure-invariant direct methods
Least-squares matrix: full	Secondary atom site location: difference Fourier map
*R*[*F*^2^ > 2σ(*F*^2^)] = 0.053	Hydrogen site location: inferred from neighbouring sites
*wR*(*F*^2^) = 0.128	H atoms treated by a mixture of independent and constrained refinement
*S* = 1.03	*w* = 1/[σ^2^(*F*_o_^2^) + (0.0528*P*)^2^ + 0.223*P*] where *P* = (*F*_o_^2^ + 2*F*_c_^2^)/3
3832 reflections	(Δ/σ)_max_ = 0.005
202 parameters	Δρ_max_ = 0.26 e Å^−^^3^
0 restraints	Δρ_min_ = −0.23 e Å^−^^3^

### Special details

**Table:**

Experimental. shape of crystal determined optically
Geometry. All e.s.d.'s (except the e.s.d. in the dihedral angle between two l.s. planes) are estimated using the full covariance matrix. The cell e.s.d.'s are taken into account individually in the estimation of e.s.d.'s in distances, angles and torsion angles; correlations between e.s.d.'s in cell parameters are only used when they are defined by crystal symmetry. An approximate (isotropic) treatment of cell e.s.d.'s is used for estimating e.s.d.'s involving l.s. planes.
Refinement. Refinement of *F*^2^ against ALL reflections. The weighted *R*-factor *wR* and goodness of fit *S* are based on *F*^2^, conventional *R*-factors *R* are based on *F*, with *F* set to zero for negative *F*^2^. The threshold expression of *F*^2^ > σ(*F*^2^) is used only for calculating *R*-factors(gt) *etc*. and is not relevant to the choice of reflections for refinement. *R*-factors based on *F*^2^ are statistically about twice as large as those based on *F*, and *R*- factors based on ALL data will be even larger.

### Fractional atomic coordinates and isotropic or equivalent isotropic displacement parameters (Å^2^)

**Table:**

	*x*	*y*	*z*	*U*_iso_*/*U*_eq_	
C1	0.2544 (3)	0.5115 (3)	0.51143 (14)	0.0381 (4)	
C2	0.2249 (3)	0.5628 (3)	0.40645 (15)	0.0509 (5)	
H2	0.2076	0.4892	0.3465	0.061*	
C3	0.2213 (4)	0.7226 (3)	0.39147 (18)	0.0609 (6)	
H3	0.2007	0.7575	0.3212	0.073*	
C4	0.2482 (4)	0.8319 (3)	0.48040 (19)	0.0597 (6)	
H4	0.2457	0.9404	0.4700	0.072*	
C5	0.2790 (3)	0.7805 (3)	0.58510 (17)	0.0479 (5)	
H5	0.2977	0.8559	0.6442	0.057*	
C6	0.2826 (3)	0.6186 (2)	0.60429 (14)	0.0366 (4)	
C7	0.3227 (3)	0.5732 (3)	0.71685 (14)	0.0406 (4)	
H7	0.388 (3)	0.492 (3)	0.7285 (18)	0.052 (6)*	
C8	0.2886 (3)	0.6664 (2)	0.99057 (14)	0.0372 (4)	
C9	0.1339 (3)	0.8556 (2)	1.06241 (14)	0.0375 (4)	
C10	0.2729 (3)	0.9819 (3)	1.12422 (16)	0.0469 (5)	
H10	0.4023	1.0094	1.1118	0.056*	
C11	0.2178 (4)	1.0678 (3)	1.20523 (17)	0.0530 (5)	
H11	0.3108	1.1530	1.2475	0.064*	
C12	0.0278 (4)	1.0282 (3)	1.22337 (17)	0.0517 (5)	
H12	−0.0080	1.0859	1.2782	0.062*	
C13	−0.1108 (3)	0.9032 (3)	1.16084 (18)	0.0514 (5)	
H13	−0.2402	0.8768	1.1732	0.062*	
C14	−0.0578 (3)	0.8164 (3)	1.07917 (16)	0.0441 (5)	
H14	−0.1512	0.7325	1.0362	0.053*	
O1	0.2599 (4)	0.2612 (3)	0.43801 (14)	0.0824 (6)	
O2	0.2395 (4)	0.2728 (2)	0.60884 (14)	0.0829 (6)	
N1	0.2507 (3)	0.3363 (2)	0.52080 (13)	0.0453 (4)	
N2	0.2793 (2)	0.6465 (2)	0.79714 (12)	0.0395 (4)	
N3	0.3341 (3)	0.6036 (2)	0.89882 (12)	0.0433 (4)	
H3B	0.411 (3)	0.546 (3)	0.9056 (18)	0.042 (6)*	
N4	0.1877 (3)	0.7690 (2)	0.97626 (13)	0.0469 (4)	
H4B	0.150 (3)	0.782 (3)	0.916 (2)	0.053 (7)*	
S1	0.35639 (10)	0.60896 (8)	1.11356 (4)	0.05372 (19)	

### Atomic displacement parameters (Å^2^)

**Table:**

	*U*^11^	*U*^22^	*U*^33^	*U*^12^	*U*^13^	*U*^23^
C1	0.0429 (10)	0.0444 (11)	0.0292 (8)	0.0173 (9)	0.0053 (7)	−0.0006 (7)
C2	0.0620 (14)	0.0657 (15)	0.0267 (8)	0.0237 (12)	0.0059 (8)	0.0002 (9)
C3	0.0796 (17)	0.0730 (17)	0.0355 (10)	0.0323 (14)	0.0075 (10)	0.0168 (10)
C4	0.0784 (17)	0.0517 (13)	0.0568 (13)	0.0310 (13)	0.0111 (12)	0.0180 (11)
C5	0.0622 (14)	0.0456 (12)	0.0411 (10)	0.0246 (11)	0.0072 (9)	0.0003 (9)
C6	0.0413 (10)	0.0440 (10)	0.0278 (8)	0.0185 (9)	0.0049 (7)	−0.0004 (7)
C7	0.0536 (12)	0.0448 (11)	0.0295 (8)	0.0255 (10)	−0.0008 (8)	−0.0045 (7)
C8	0.0494 (11)	0.0370 (10)	0.0281 (8)	0.0190 (9)	0.0010 (7)	−0.0027 (7)
C9	0.0547 (12)	0.0367 (10)	0.0281 (8)	0.0247 (9)	0.0037 (7)	0.0017 (7)
C10	0.0467 (12)	0.0527 (13)	0.0433 (10)	0.0194 (10)	0.0061 (9)	−0.0046 (9)
C11	0.0640 (15)	0.0501 (13)	0.0444 (11)	0.0199 (11)	0.0005 (10)	−0.0139 (9)
C12	0.0698 (15)	0.0571 (13)	0.0395 (10)	0.0352 (12)	0.0114 (10)	−0.0032 (9)
C13	0.0521 (13)	0.0590 (14)	0.0493 (11)	0.0248 (11)	0.0152 (10)	0.0095 (10)
C14	0.0541 (12)	0.0363 (10)	0.0407 (10)	0.0147 (9)	0.0008 (9)	0.0021 (8)
O1	0.1373 (19)	0.0725 (12)	0.0507 (10)	0.0532 (13)	0.0082 (10)	−0.0205 (9)
O2	0.155 (2)	0.0529 (11)	0.0455 (9)	0.0410 (12)	0.0083 (11)	0.0041 (8)
N1	0.0516 (10)	0.0481 (10)	0.0377 (8)	0.0193 (8)	0.0037 (7)	−0.0076 (7)
N2	0.0534 (10)	0.0419 (9)	0.0275 (7)	0.0222 (8)	0.0021 (6)	−0.0007 (6)
N3	0.0664 (12)	0.0493 (10)	0.0259 (7)	0.0359 (10)	−0.0009 (7)	−0.0034 (6)
N4	0.0751 (13)	0.0546 (11)	0.0257 (7)	0.0425 (10)	−0.0017 (7)	−0.0034 (7)
S1	0.0844 (4)	0.0706 (4)	0.0259 (2)	0.0526 (3)	0.0011 (2)	0.0008 (2)

### Geometric parameters (Å, º)

**Table:**

C1—C2	1.385 (3)	C9—C14	1.374 (3)
C1—C6	1.405 (2)	C9—C10	1.377 (3)
C1—N1	1.472 (3)	C9—N4	1.432 (2)
C2—C3	1.369 (3)	C10—C11	1.386 (3)
C2—H2	0.9300	C10—H10	0.9300
C3—C4	1.379 (3)	C11—C12	1.365 (3)
C3—H3	0.9300	C11—H11	0.9300
C4—C5	1.384 (3)	C12—C13	1.374 (3)
C4—H4	0.9300	C12—H12	0.9300
C5—C6	1.394 (3)	C13—C14	1.389 (3)
C5—H5	0.9300	C13—H13	0.9300
C6—C7	1.470 (2)	C14—H14	0.9300
C7—N2	1.273 (2)	O1—N1	1.209 (2)
C7—H7	0.97 (2)	O2—N1	1.202 (2)
C8—N4	1.327 (2)	N2—N3	1.371 (2)
C8—N3	1.349 (2)	N3—H3B	0.87 (2)
C8—S1	1.6799 (18)	N4—H4B	0.79 (3)
			
C2—C1—C6	122.28 (19)	C10—C9—N4	120.05 (19)
C2—C1—N1	116.16 (17)	C9—C10—C11	119.3 (2)
C6—C1—N1	121.55 (16)	C9—C10—H10	120.4
C3—C2—C1	119.5 (2)	C11—C10—H10	120.4
C3—C2—H2	120.2	C12—C11—C10	120.4 (2)
C1—C2—H2	120.2	C12—C11—H11	119.8
C2—C3—C4	120.11 (19)	C10—C11—H11	119.8
C2—C3—H3	119.9	C11—C12—C13	120.19 (19)
C4—C3—H3	119.9	C11—C12—H12	119.9
C3—C4—C5	120.2 (2)	C13—C12—H12	119.9
C3—C4—H4	119.9	C12—C13—C14	120.0 (2)
C5—C4—H4	119.9	C12—C13—H13	120.0
C4—C5—C6	121.7 (2)	C14—C13—H13	120.0
C4—C5—H5	119.1	C9—C14—C13	119.4 (2)
C6—C5—H5	119.1	C9—C14—H14	120.3
C5—C6—C1	116.22 (17)	C13—C14—H14	120.3
C5—C6—C7	119.24 (17)	O2—N1—O1	122.1 (2)
C1—C6—C7	124.48 (17)	O2—N1—C1	119.95 (16)
N2—C7—C6	119.59 (17)	O1—N1—C1	117.90 (18)
N2—C7—H7	121.3 (13)	C7—N2—N3	115.10 (15)
C6—C7—H7	119.1 (13)	C8—N3—N2	120.88 (16)
N4—C8—N3	116.38 (16)	C8—N3—H3B	118.3 (14)
N4—C8—S1	124.22 (13)	N2—N3—H3B	120.3 (14)
N3—C8—S1	119.39 (14)	C8—N4—C9	125.10 (16)
C14—C9—C10	120.63 (17)	C8—N4—H4B	118.8 (17)
C14—C9—N4	119.25 (19)	C9—N4—H4B	116.1 (17)
			
C6—C1—C2—C3	−0.5 (3)	C11—C12—C13—C14	0.2 (3)
N1—C1—C2—C3	178.3 (2)	C10—C9—C14—C13	−1.3 (3)
C1—C2—C3—C4	0.4 (4)	N4—C9—C14—C13	−178.36 (18)
C2—C3—C4—C5	0.0 (4)	C12—C13—C14—C9	0.6 (3)
C3—C4—C5—C6	−0.4 (4)	C2—C1—N1—O2	−166.3 (2)
C4—C5—C6—C1	0.4 (3)	C6—C1—N1—O2	12.5 (3)
C4—C5—C6—C7	177.9 (2)	C2—C1—N1—O1	14.0 (3)
C2—C1—C6—C5	0.1 (3)	C6—C1—N1—O1	−167.2 (2)
N1—C1—C6—C5	−178.62 (18)	C6—C7—N2—N3	−175.80 (18)
C2—C1—C6—C7	−177.3 (2)	N4—C8—N3—N2	0.6 (3)
N1—C1—C6—C7	4.0 (3)	S1—C8—N3—N2	179.32 (15)
C5—C6—C7—N2	27.7 (3)	C7—N2—N3—C8	−176.8 (2)
C1—C6—C7—N2	−155.0 (2)	N3—C8—N4—C9	−176.5 (2)
C14—C9—C10—C11	1.1 (3)	S1—C8—N4—C9	4.9 (3)
N4—C9—C10—C11	178.15 (19)	C14—C9—N4—C8	−115.2 (2)
C9—C10—C11—C12	−0.2 (3)	C10—C9—N4—C8	67.7 (3)
C10—C11—C12—C13	−0.4 (4)		

### Hydrogen-bond geometry (Å, º)

**Table:**

*D*—H···*A*	*D*—H	H···*A*	*D*···*A*	*D*—H···*A*
N3—H3*B*···S1^i^	0.87 (2)	2.51 (2)	3.3664 (18)	168.8 (19)
N4—H4*B*···N2	0.79 (3)	2.29 (2)	2.642 (2)	108 (2)
C7—H7···O2	0.97 (2)	2.26 (2)	2.703 (3)	106.5 (16)
C11—H11···O1^ii^	0.93	2.60	3.214 (3)	125

Symmetry codes: (i) −*x*+1, −*y*+1, −*z*+2; (ii) *x*, *y*+1, *z*+1.
